# Comparative Assessment of Medicinal Plant Utilization among Balti and Shina Communities in the Periphery of Deosai National Park, Pakistan

**DOI:** 10.3390/biology10050434

**Published:** 2021-05-14

**Authors:** Zaheer Abbas, Shazia Kousar, Muhammad Abdul Aziz, Andrea Pieroni, Ali Abdullah Aldosari, Rainer W. Bussmann, Ghulam Raza, Arshad Mehmood Abbasi

**Affiliations:** 1 Department of Botany, University of Education Lahore, Lahore 54770, Pakistan; zaheer.abbas@ue.educ.pk; 2 Department of Botany, University of Karachi, Karachi 75270, Pakistan; kousar110@hotmail.com; 3 University of Gastronomic Sciences, 12042 Pollenzo, Italy; m.aziz@studenti.unisg.it (M.A.A.); a.pieroni@unisg.it (A.P.); 4 Department of Medical Analysis, Tishk International University, Erbil 44001, Iraq; 5 Geography Department, King Saud University, Riyadh 11451, Saudi Arabia; adosari@ksu.edu.sa; 6 Department of Ethnobotany, Institute of Botany, Ilia State University, 0105 Tbilisi, Georgia; rainer.bussmann@iliauni.edu.ge; 7 Department of Biological Sciences, University of Baltistan, Skardu 15100, Pakistan; ghulam.raza@uobs.edu.pk; 8 Department of Environmental Sciences, COMSATS University Islamabad, Abbottabad 22060, Pakistan

**Keywords:** ethnobotany, cross-culture, medicinal plants, Deosai, Pakistan, Himalaya

## Abstract

**Simple Summary:**

Traditional ecological knowledge is a key contributor to environmental sustainability; therefore, it is essential to identify and preserve this biocultural heritage. We documented traditional uses of plant species among the two marginalized communities, namely Baltis and Shinas, living in Deosai National Park, western Himalayas, Pakistan, using random and purposive sampling techniques targeting middle- and old-aged informants. In total, 47 medicinal plant species were recorded, which were cited by both Baltis and Shinas (42 and 38 plant species, respectively) to treat various diseases. Considerable homo- and heterogeneities were noted in vernacular names, plant part(s) used, drug formulation, and administration. *Ribes alpestre*, *Aconitum violaceum*, *Delphinium brunonianum*, *Thymus linearis*, and *Swertia petiolata* were the highly utilized species. In addition, medicinal uses of *Allardia tomentosa*, *A. tridactylites*, *Jurinea dolomiaea*, and *Gallium boreale* were reported for the first time from this region. Both Balti and Shina communities retain substantial biocultural and ethnological diversity, which has been reflected in the present survey.

**Abstract:**

Traditional ecological knowledge, linguistic, and sociocultural perspectives are key contributors to environmental sustainability. Therefore, it is essential to identify and preserve this biocultural heritage, especially that of indigenous communities and minorities. We conducted an ethnobotanical survey to document the plant species used by the Balti and Shina communities living in the buffer zone of Deosai National Park (DNP), western Himalayas, Pakistan. A combination of random and purposive sampling techniques was adapted, targeting middle- and old-aged informants. A total of 46 semi-structured interviews were conducted and the gathered data were evaluated using relative frequency of citation (RFC) and through comparison with the ethnomedicinal literature. In total, 47 medicinal plant species belonging to 42 genera and 23 families were recorded. Baltis and Shinas cited 42 and 38 plant species, respectively, that were used to treat various diseases. About 60% of species were common among both communities, but 27.7% and 12.8% were exclusive to Baltis and Shinas, respectively. Considerable heterogeneity was noted in vernacular names, plant part(s) used, preparation, and administration. *Ribes alpestre*, *Aconitum violaceum*, *Delphinium brunonianum*, *Thymus linearis*, and *Swertia petiolata* were highly utilized species having RFCs > 50. In addition, 46% of medicinal uses, specifically that of *Allardia tomentosa*, *A. tridactylites*, *Jurinea dolomiaea*, and *Gallium boreale*, were reported for the first time from the region. Cross-cultural analysis revealed sociocultural gaps between both groups. Relatively, Baltis retained more ethnomedicinal knowledge and their traditional medicinal system is more closely associated with traditional Tibetan medicine. Generally, Balti and Shina communities retain substantial biocultural and ethnological diversity, which has been reflected in the present study. Our findings underline the importance and need for sustainable utilization of natural resources, specifically the plant species of this region. However, an in-depth ethnobotanical investigation may underpin the holistic comparative medical ethnobotany of the entire region.

## 1. Introduction

Humans and nature have been inextricably linked with each other throughout human history. Every culture has utilized natural resources in order to satisfy their needs and strived to maintain the integrity of the local ecosystem [1]. Traditional ecological knowledge (TEK) about biodiversity is an important element in the daily life of any cultural group and has always been influenced by changing socio-ecological conditions. TEK of medicinal plants has greatly contributed to local healing systems among many societies across the globe [2].

Globalization has made us progressively aware of both the unity and the diversity of our planet. Although a huge global interconnectedness has led us to remarkable cultural, political, and economic integration, the risk of linguistic and cultural homogenization has also increased. Similarly, the fast-growing problems of biodiversity loss, habitat degradation, and the erosion of indigenous/traditional ecological knowledge represent threats to the living planet and have drawn the attention of the global community toward a possible “extinction crisis.” Reports have shown the extinction of 11,000–36,000 species each year and in many areas of the world species richness is declining below the critical threshold that is needed to ensure the viability of natural ecosystems [3]. UNESCO has estimated that half of the 6000 plus languages spoken today will disappear by the end of the century. As defined by Maffi [4], the concept of biocultural diversity is “the diversity of life in all its manifestations—biological, cultural and linguistic”, which are interrelated within a complex socio-ecological adaptive system. Several initiatives have been attempted at a global scale to stem the loss of biodiversity, but unfortunately, thus far, such adopted conservation measures to protect biodiversity have not produce very promising results. The Convention on Biological Diversity, for example, has played a very passive role despite its wide ratification and large investment [5]. Consequently, the slow rate of progress has led the international community to search for alternative solutions. From a range of different options, two are receiving considerable attention: the new conservation science approach and the half earth approach. However, their arguments are apparently pitted against one another, presenting opposing visions of policy tools, focal locations, and value orientations. Since conservation is a social process, many researchers support biocultural approaches as they represent a more pluralistic and collaborative strategy that involves all stakeholders to achieve sustainable conservation goals. Biocultural approaches strongly recognize TEK as the main pillar that helps in implementing conservation strategies, and thus, we believe that ethnobotanical field studies can help to protect disappearing TEK and provide a foundation for future conservation efforts [6].

Pakistan is home to several thousand plant species, and the northern belt of the country, in particular, is considered a reservoir of important plant species, which are used for different purposes. Local communities have retained considerable knowledge of these natural resources [7]. In recent times, globalization and modernization have been reducing the interest of, especially, younger generations of local communities in traditional healing systems. This is threatening TEK, in addition to undermining the importance of local plant resources. However, in remote and mountain areas, people still use medicinal plants to treat a wide variety of illnesses [8]. Deosai National Park is situated in the west of the Tibetan Plateau in Pakistan. The area is inhabited by various linguistic minorities who have retained important traditional knowledge on local medicinal plants and ecosystem management. Deosai National Park has rarely been explored in terms of traditional medicinal knowledge, despite its rich biodiversity. While some ethnobotanical studies have been conducted in neighboring areas [9,10], a cross-cultural comparative analysis has not yet been carried out. Living in a multicultural society, each ethnic group strives to maintain its cultural identity while achieving better livelihoods, allowing us to assess the fascinating question as to how plant knowledge changes across time and space [11]. Research has shown that cultural and linguistic edges have always played an important role in articulating human perspectives on the diverse biological world, and, more importantly, that language contains a wealth of information about nature, including plants [12]. The current study was undertaken among Balti and Shina communities living in a few remote mountain villages of Deosai National Park. Balti is a native Tibetan language, while Shina is a Dardic language that is widespread in the territory of Gilgit-Baltistan. The study aimed to record the traditional ecological knowledge of wild medicinal plants among the two considered linguistic groups to provide a baseline for future conservation programs in the region.

The main objectives of the study were as follows:

(a) to record the phytonyms and traditional knowledge of medicinal plants gathered among the two linguistic communities;
(b) to make a cross-cultural comparison of the gathered data between the two considered groups in order to understand the sociocultural adaptations that these groups have undergone;
(c) to compare the gathered data with the existing Tibetan ethnomedicinal literature.

## 2. Materials and Methods

### 2.1. Study Area and Targeted Communities

**Figure 1:**
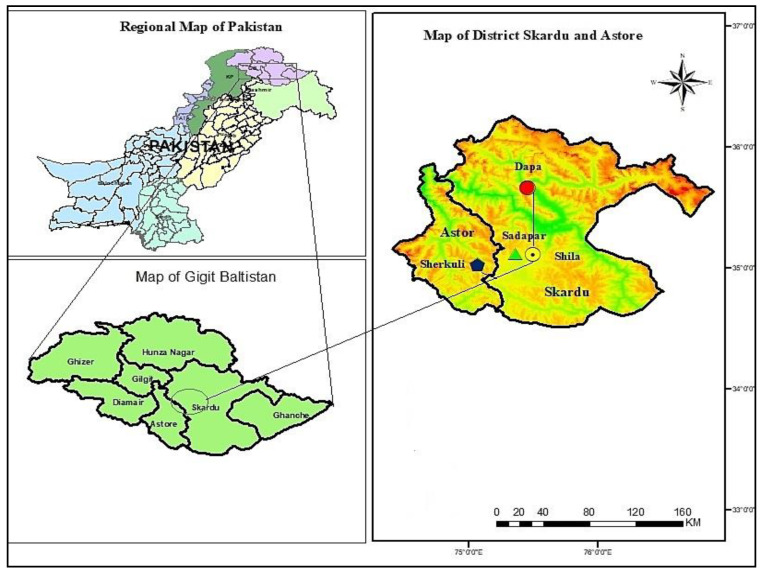
Map of the study area.

Deosai National Park (DNP) is an alpine highland in the western Himalayan range of Pakistan. It is located at 350.02′ N, 0750.25′ E, covering parts of three districts, i.e., Astor, Kharmang, and Skardu (Figure 1), with an area of 1400 km^2^ and an average elevation of 4114 m.a.s.l. [8]. Locally, Deosai is known as Ghbiarsa (Ghbiar-Summer, Sa-Land/the land of summer). Geologically, the Deosai Plateau is characterized by the Ladakh batholith and volcanic rock series of the late Jurassic and Cretaceous periods along with Quaternary glacio-fluvial and alluvial deposits [13,14]. The melting snow forms channels of alpine streams, such as Chogho Chu (Bara Pani), Naqpo Chu (Kala Pani), and Shatong Chu (Shatong Nala), eventually transforming into rivers, i.e., Sadpara, Shila, Katisho, Shingo, Gultari, and Chillam, that irrigate agriculture lands in the buffer zone. In the peripheral zone of DNP, Shila, Dapa, Shingo, Gultari, Chillam, and Sadpara are the main villages. Based on linguistic and cultural diversity, two main communities, namely Baltis and Shinas, live in four different villages: Shila and Dapa (Baltis) and Sadpara and Sherkuli (Shinas), which were targeted to collect ethnomedicinal information. The Balti and Shina populations are different in their origin, physique, culture, language, pronunciation, and behavior [15,16].

**Figure 2:**
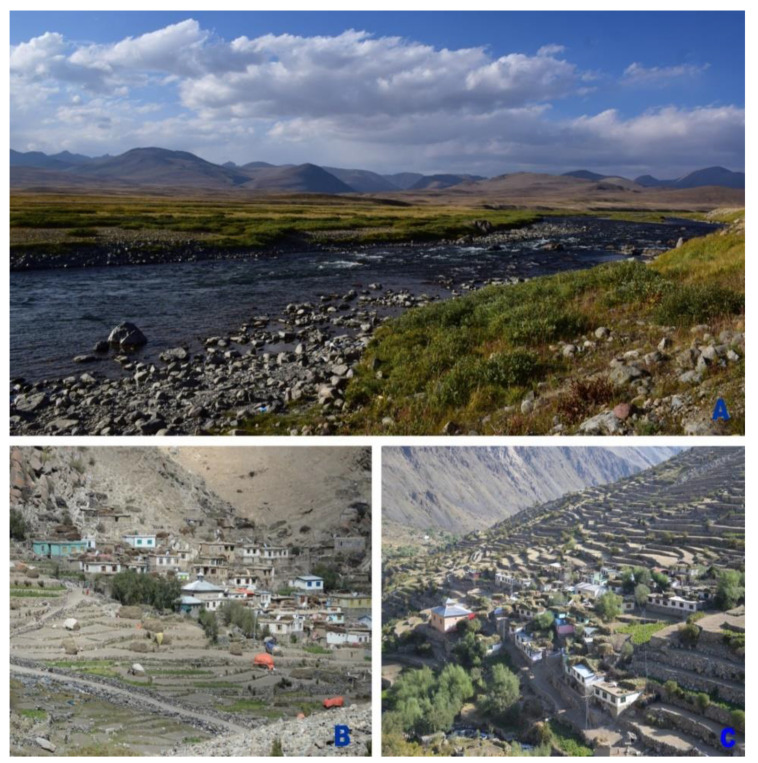
Panoramic view of Deosai National Park (**A**), Shila and Dapa communities (**B**,**C**).

Panoramic views of Deosai National Park and the settlements of Shila and Dapa are shown in Figure 2, while the characteristics of the targeted localities and study participants are given in Table 1. The village of Shila, formerly known as Shingla (Shing-wood, La-track/track of wood), is located on the left bank of the Shila River at an elevation of 3252 m.a.s.l. This village is comprised of only 70 households of the Balti community. Baltis living in Shila are Noorbakhsh, a branch of Shia Muslims. Being Purki (belong to Purik/Purig/Kargil, Ladakh India), Baltis belong to a single clan but those living in Shila have different communication styles and accents, compared to the Baltis living at lower altitudes in Gilgit and Baltistan. Baltis in Shila are marginalized, poor, and lack medical, educational, and communication facilities. They are mostly engaged in mountain agropastoral systems. Potato, pea, and barley are their main crops. 

Dapa village is situated on the right bank of the River Katisho and is the last and highest village of Katisho Valley at an elevation of 3886 m.a.s.l. Dapa is home to 180 households and its inhabitants are Balti, by origin, who migrated from Ladakh, India. Dapa was once the most productive village for wheat, and people from other parts of Baltistan, particularly from lower Kharmang, used to go there to exchange goods (sugar, salt, tea, dry fruits) for wheat grains. However, now the Baltis in Dapa are engaged in small-scale farming (pea, potato, wheat) and herding, but there is an increasing trend of migration toward the town of Skardu, lowland cities, and abroad (Middle East) in search of jobs. In Dapa, Balti women are more laborious compared to men. They are engaged in demanding mountain agriculture, constructing and managing impressive field terraces. Sadpara is located at 2350 m.a.s.l. in the northern entrance of DNP. Sadpara is dominated by the Shina community residing on both sides of the Sadpara River, with a negligible minority of Balti people. Inhabitants of Sadpara are bilingual and speak both Balti and Shina, and even sometimes blend the two languages. Pottery production, trekking, herding, contractual government work, and small-scale farming are the main subsistence activities of the Sadpara people. Sherkuli is the highest village of the district Astor, toward western side of DNP along the right bank of the River Chillam, at an altitude of 3355 m a.s.l. Sherkuli comprises 150 households that are Shin Dard Muslim. The Astori Shina dialect is closely related to the neighboring communities of the Chilas, Darel, and Tangir valleys. The population uses Urdu as a lingua franca to communicate with people of other languages. Mostly these people engage in herding and farming.

The migration of Shinas from Sherkuli Balti dominated areas of Skardu and other cities in search of business opportunities, jobs, better educational facilities, and health services is high. In addition, a substantial number of Astori migrants also migrate from Chillam to Skardu to join their relatives and many have already settled in Skardu town. Informal communications during such visits could be a potential source of knowledge transfer that leads to homogeneity in traditional knowledge. Similarly, herders and livestock butchers visiting both places for business purposes, particularly at Eid festivals, also share such information with each other. One of the potential factors in merging local knowledge is the shared agropastoral practices, including the exchange of seeds for domestic alteration, the exchange of male and female livestock, or the exchange of livestock of different domestic varieties or breeders.

### 2.2. Field Study

**Figure 3:**
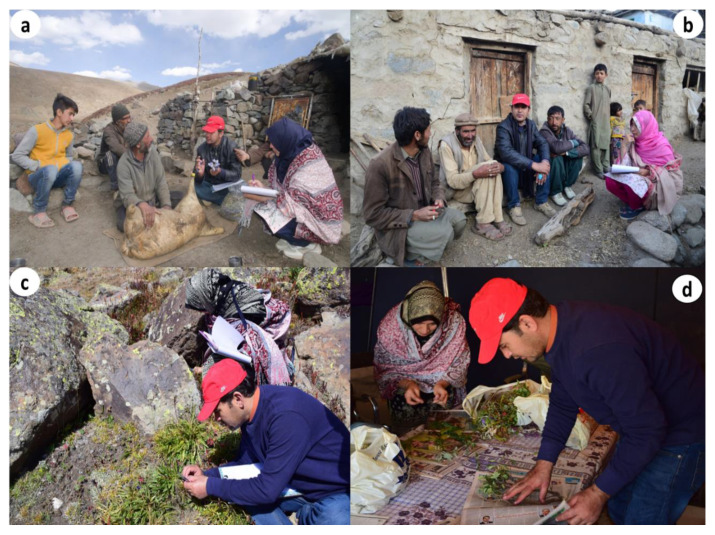
Field activities: (**a**,**b**) first two authors conducting interviews, (**c**) collection and identification of plants, and (**d**) sorting and pressing of plant specimens.

An ethnobotanical field survey was conducted in late summer 2019, in the four aforementioned villages of Dapa and Shila (Balti community) and Sadpara and Sherkuli (Shina community) of the DNP buffer zone (Figure 3). The Code of Ethics of the International Society of Ethnobiology [17] was strictly followed during the field survey. A combination of random and purposive sampling techniques as suggested by [18,19] was adapted to target middle- and old-aged people (mainly farmers and shepherds).

Verbal informed consent was obtained from informants prior to each interview. Semi-structured interviews, as reported previously [20,21], were conducted by the first two authors in the participants’ houses, gathering places, and with shepherds in alpine pastures (Figure 3; the participants shown in the figure consented to publication). The interviews were conducted in Balti, Shina, and Urdu languages, and information was subsequently translated into English. The respondents were asked to share information related to medicinal plants, including vernacular names, used plant part(s), drug preparation, and diseases treated. During the interviews, participants were also asked to show preserved plant material and recorded information in writing (if available).

Plants were collected in the field and identified by local respondents/guides. Taxonomic nomenclature was mainly based on the Flora of Pakistan [22], Flora of Deosai plains [23], and Flora of China (http://www.efloras.org/flora_page.aspx?flora_id=2 (accessed on 11 March 2021)). Plant identification was confirmed using the Plant List [24] and Angiosperm Phylogeny [25]. The identified specimens were assigned voucher numbers, properly labeled, stamped, and stored at the head office of Central Karakorum National Park (CKNP), Skardu-Baltistan.

### 2.3. Data Analysis

The collected data were organized in MS Excel. Frequency of citation (FC) is the total number of respondents reporting the medicinal use of a species. Relative frequency of citation (RFC) was calculated to determine the importance of every medicinal taxon in the given communities using the formula, as described earlier [26]:

(1) RFCs=FCs/N

where, FC is the number of informants who mentioned the use of a plant species, and N is the total number of informants.

Venn diagrams were generated to show the possible associations regarding plant resource utilization to treat various diseases between Balti and Shina communities. Data were categorized into two sets based on target communities, and comparative analysis was conducted through proportional Venn diagrams, using freely available software (http://bioinformatics.psb.ugent.be/webtools/Venn/ (accessed on 20 April 2021)).

The data of all cited species with their reported medicinal uses were compared and cross-checked with the available ethnomedicinal literature of neighboring areas, i.e., Gilgit-Baltistan, Kohistan, Hazara, Azad Jammu and Kashmir, and the Ladakh region of India, published from 2000 to 2020 [27,28,29,30,31,32,33,34,35,36,37,38,39,40,41]. In addition, the ethnobotanical literature of Tibetan communities in China, India, Nepal, and Pakistan was also considered to find ethnomedicinal linkages.

## 3. Results and Discussion 

### 3.1. Medicinal Plants and Their Uses

We documented the medicinal uses of 47 plant species used by the inhabitants of Balti and Shina communities. Each plant species was documented with its botanical name and voucher number, family, local name, habit, part(s) used, disease cured, mode of administration, and relative frequency of citation (Table 2). It was noted that both communities hold extensive traditional knowledge about wild herbaceous medicinal plants belonging, especially, to Asteraceae, Apiaceae, Lamiaceae, and Polygonaceae. Local inhabitants gathered all the plant species from the wild, except for *Fagopyrum esculentum*, which was cultivated at higher elevations.

Leaves were the most commonly used plant parts, followed by flowers, roots, and seeds. The frequent use of leaves could be attributed to the fact that they contain more active phytochemicals, compared to other parts of a plant. It is worth mentioning that the access and availability of a specific plant part is also a main driver for its selection. The findings of the current study demonstrate that Baltis frequently used leaves and flowers, which might be due to the fact that their ancestors had strong religious affiliations with Buddhism. Additionally, in Buddhism, flowers and fresh plant parts carry strong spiritual significance. Therefore, this practice was possibly passed down and maintained by their Balti decedents, for whom it is an integral part of their traditional health care system. Among both communities, the majority of the reported plant species were commonly processed into decoctions, which have been reported as one of the best approaches to extract beneficial secondary metabolites [42,43].

Most of the traditional remedies (from 37 species) were taken orally. Gastro-intestinal ailments were among the most treated diseases using more than 20 species, followed by skin diseases and respiratory tract disorders (11 species each), and hepato-pancreatic disorders (5 species) The limited health facilities in the studied areas may be one of the main potential factors for the aforementioned pathological scenario; in addition, as reported earlier [43,44], such disorders are common in the inhabitants of the mountain communities. Studies conducted in the Shina communities of Gilgit [45,46] revealed profound differences in medicinal plant utilization compared to the Shinas in our study area. For instance, there is a difference in the vernacular name of *Betula utilis*, i.e., Jowzee (Shinas in other areas) and Jongi (Shinas in the current study area). An extract prepared from the bark of this species has different uses, as it is used to treat earache in the Shina speaking Haramosh Valley of Gilgit [47], while the bark powder of the same species is used for backache and leg pain by Shinas in DNP. Similarly, the seeds of *Bistorta affinis* (Chughuy in Gilgit) are used to treat dysentery and urinary bladder disorder among the Shina community in Gilgit [46], but Shinas in the Skardu area of DNP use the rhizome of the same species to treat diarrhoea and fever. In addition, there is variation in the vernacular name of this plant species among Shina-peaking communities of Gilgit and DNP, i.e., it is known as Chughuy in Gilgit, but Chomoi in the Shina language spoken by Shinas in DNP. Variation in nomenclature and ethnomedicinal uses of the same plant species among the same communities living in the different areas reveals heterogeneity in their traditional knowledge. This might be due to variation in their point of origin and migratory routes, perceptions about plant resource utilization, and interactions with different linguistic and ethnic groups in the area of their settlement.

**Figure 4:**
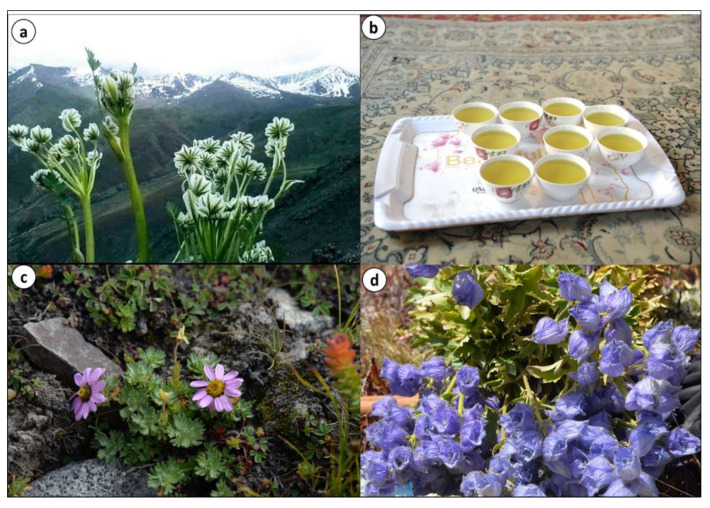
(**a**) *Pleurospermum candolleii*, (**b**) *P. candolleii* tea exclusive to Shila village, (**c**) *Allardia tridactylites*, and (**d**) *Delphinium brunonianum* a common medicinal plant of Baltis and Shinas.

**Figure 5:**
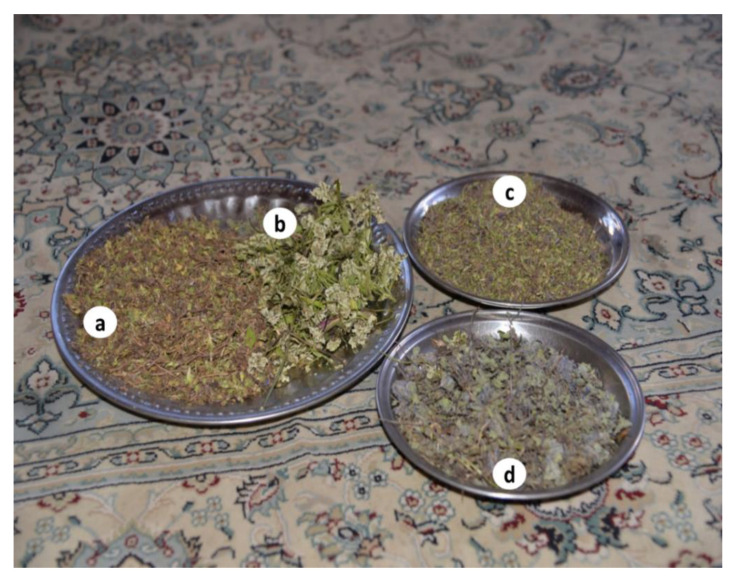
Locally gathered medicinal herbs: (**a**) Allardia tridactylites, (**b**) Pleurospermum candollei, (**c**) Thymus linearis, and (**d**) Nepeta leucolaena.

During the field survey, it was observed that some plant species, namely *Thymus linearis*, *Arnebia benthamii*, *Aconitum violaceum*, and *Carum carvi*, are also sold in local markets, whereas *Allardia tridactylites*, *Nepeta leucolaena*, and *Pleurospermum candollei* are dried and stored at home for use in the offseason (Figure 4). Various plant species are collected from higher elevations by shepherds for both medicinal and food purposes, among these *Delphinium brunonianum* and *Thymus linearis* are common (Figure 5). However, the collection, storage, and selling of plants are on a small scale, and therefore do not make a considerable contribution to the economic growth of local communities. The sale of herbs in the whole region is rough and random, as there is no proper marketing chain where people might properly sell their collected materials. The prize of locally cultivated plants is at the mercy of bargainers in the markets. Poor people living at high elevations seasonally carry their collected plants to town for market circulation and sell at very low prices. Grazing, trampling, and unsustainable collection are the main threats to medicinal plants. For now, the health infrastructure is not much developed in villages, but allopathic drugs are widely used that will erode ethnomedicinal knowledge and the traditional use of local flora.

Measured values of the relative frequency of citation (RFC) of the reported species ranged from 84.78 to 13.04 (Table 2), which indicates a high validity of medicinal uses of the reported species. With reference to RFC values, the recorded plant species were divided into three groups: high (70–90), moderate (50–70), and low (below 50). The first group included six species with the highest RFCs: *Ribes alpestre* (84.78), *Aconitum violaceum* (78.26), *Delphinium brunonianum* (78.26, Figure 4d), *Thymus linearis* (71.73), *Taraxacum officinale* (71.73), and *Swertia petiolata* (71.73). *Ribes alpestre* is common in wastelands and among field terraces and its fruit is edible [48]. Previous studies from Shigar Valley, Karakorum [46] and the Diamer area of the Himalayas [45] reported RFCs of 15 and 26, respectively, for *R. alpestre*, which also underline its therapeutic importance.

*Aconitum violaceum* is a common medicinal plant species of Deosai meadows [9,49], and its ethnomedicinal uses are well established throughout Gilgit and Baltistan. Similarly, *Delphinium brunonianum* is a commonly utilized medicinal taxon throughout the region. It is extensively used by Baltis and Shinas for baldness and pneumonia, respectively. *Thymus linearis* is a high ecological amplitude species of northern mountain systems, which is collected by local inhabitants for domestic use as a medicinal tea. This species is also available in local markets of both Astor and Skardu towns. *Swertia petiolata* is a rare species and is found along alpine streams. Medicinal uses of this species among Balti and Shina communities were comparable to those previously reported by Bano et al. [49] from Skardu Valley.

The second group comprised 18 species with RFCs values ranging from 50 to 70. Among these, *Allium carolinianum*, *Cousinia thomsonii*, *Mentha royleana*, *Nepeta leucolaena*, and *Bergenia stracheyi* are commonly utilized species. The current status with respect to RFCs revealed that traditional uses of these plant species are still maintained but may be gradually vanishing. It is interesting to note that species with RFCs lower than 50 were the most numerous (23 species). Low levels of RFCs indicate that traditional knowledge of these species, including *Chenopodium foliosum*, *Bistorta affinis*, *Rubia cordifolia*, *Betula utilis*, and *Myosotis alpestris*, is threatened.

### 3.2. Cross-Cultural Comparison

**Figure 6:**
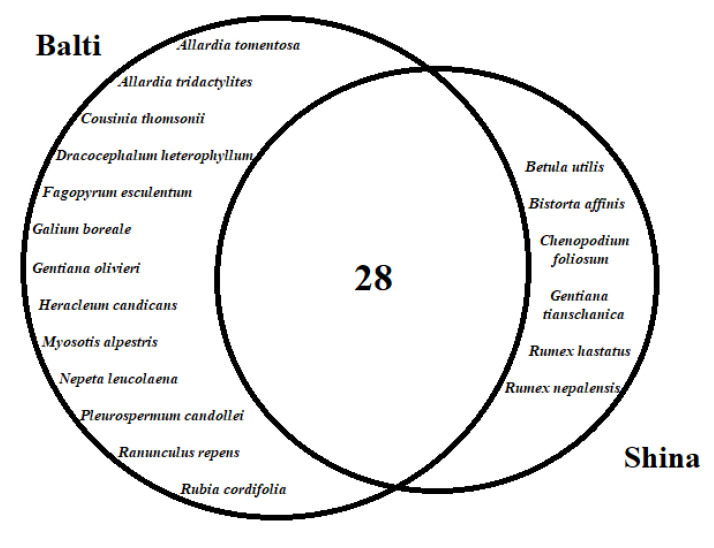
Venn diagram illustrating utilization of medicinal plant species among the two studied groups.

The cross-cultural comparison showed that more than half of the reported medicinal plants were commonly used by both studied groups (Figure 6). Relatively, Baltis retained rich traditional medicinal knowledge because they are native to the area. All plant species used by Balti people were also used by Shinas, who possibly adapted to the Balti culture after their arrival and settlement in the study area. In addition, Baltis live in high elevation areas and possess more information on the medicinal uses of high mountain plant species, which is why they exclusively prefer and use these plants. It is also important to note that since Baltis are formerly from Tibet, their traditional medicinal system is greatly influenced by the orally transmitted and written scripts of traditional Tibetan medicine.

**Figure 7:**
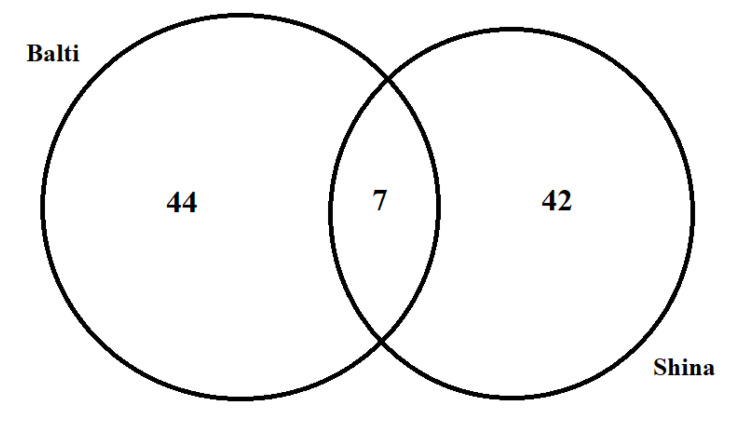
Comparative analysis of the use reports of the commonly shared medicinal species between the studied groups.

Out of 47 medicinal plant species, 28 were commonly utilized by both Balti and Shina groups, as shown in Figure 7. Comparatively, both groups showed remarkable divergences among the use reports of the commonly shared medicinal species. The dissimilarity in medicinal plants in terms of use reports may indicate certain sociocultural gaps, which in turn have prevented the sharing of traditional knowledge among the respective ethnic groups, especially since they do not intermarry (even though they share the same faith).

In the study area, the two researched groups followed different plant nomenclatures, with the exception of *Aconitum violaceum*, which was reported by both linguistic groups with the common name of “*Buma*.” This indicates an inextricable and complex network of relationships that a given cultural/linguistic group established with the surrounding flora; thus, language creates sound basis for the retention of TEK within a socio-ecological region. Moreover, the cultural isolation between the two researched groups has helped the local communities to articulate the local knowledge and retain their specific interpretations regarding the use of natural resources. The lack of commonalities in local names of the recorded species between the two groups could be due to cultural isolation which has prevented the adoption of a single and standardized phytonym for each plant species.

### 3.3. Comparison with Tibetan Ethnobotany

Ethnomedicinal uses of plant species among Balti and Shina communities were compared with more than 30 reports from the Tibetan Plateau and peripheral regions of China (Gansu, Qinghai, Yunnan, and Sichuan), Nepal (Mustang, Dolpo, and Limi), India (Ladakh, Himachal Pradesh, Uttarakhand, Sikkim, and Aurunachal Pradesh), and Pakistan (Gligit and Baltistan). Perhaps: comparative assessment revealed ethnolinguistic similarities of Tibetan populations with Muslim Bhotia of the study area [50,51]. However folk names of medicinal plants quoted in the present study were mostly different from those reported previously for the same species from other Tibetan-based regions of China, Nepal, and India [49,50,51,52], except few cases, e.g., the local name of juniper in Baltistan is “*Shukpa*” and in Ladakh it is “*Sukpa*”. Similarly, the same medicinal plants, such as *Bergenia stracheyi*, *Arnebia euchroma*, *Carum carvi*, *Peganum harmala*, *Rubia cordifolia*, *Hippophae rhamnoides*, *Tribulus terrestris*, *Rosa webbiana*, *Heracleum candicans*, *Aconitum violaceum*, *Urtica dioca*, and *Thymus linearis*, are used by other communities in the region, although a different plant part may be used and a different disease may be treated. *Arnebia euchroma*, *Thymus linearis*, *Carum carvi*, and *Urtica dioca* are also well-known medicinal herbs in other Tibetan regions. In Mustang (Nepal), the seeds of *Thymus linearis* and *Carum carvi* are used as wild condiments, while in our study areas these species are used to treat gastric ulcers [53]. Soup made from the young shoots of *Urtica dioica* (zwa) is prepared by the Tibetan community of Nepal [54], while Balti and Shina communities use the leaves of this species to alleviate constipation. Tibetan communities of Litang (Nepal) use the crushed seeds of *Carum carvi* (go snyod) as a spice named Naqpo Thalae [55], while in our study groups, this species is used to cure ulcers. The variations and homogeneity in the traditional uses of medicinal plants not only indicate a specific association between the Balti community and Tibetan people of Nepal, but also reveal their adaptation to a new environment (Baltistan region of Pakistan) after migration from their place of origin (Tibet) a long time ago. This can be further confirmed by the fact that the medicinal plant species reported by Tibetan Baltis who migrated to other mountain areas of Ladakh are similar but with different nomenclature and ethnobotanical uses. For instance, the fruit of *Rosa webbiana* (Se ba) is eaten raw, used in making jam, and mixed with chili as a condiment in Ladakh [40], but it is used as a tonic and to treat anemia and jaundice in our study. Similarly, the fruit of *Delphinium brunonianum* (bya rgod spos) is used commercially in juice production, while its flowers were reported by Baltis as an effective recipe for baldness. The roots of *Bergenia stracheyi* and *Arnebia euchroma*, the seeds of *Carum carvi*, *Peganum harmala*, and *Rubia cordifolia*, and the fruits of *Hippophae rhamnoidse* were reported to treat kidney disorders in Ladakh [39], which is entirely different from the medicinal uses of these species among Balti and Shina communities of DNP. Similarly, *Rosa webbiana* (flowers, fruits), *Tribulus terrestris* (fruits), and *Rubia cordifolia* (roots) have been reported to treat fever, cold, and cough in Ladakh [38], but Baltis and Shinas use these plant species to treat dermal infections and anemia.

In the Kargil District of Ladakh, India, Balti inhabitants used the roots of *Aconitum violaceum* to cure cough, fever, vomiting, nausea, and piles, while in our study, the same species was used for flatulence and abdominal pain. The roots of *Arnebia euchroma* have been reported to improve hair health, and to alleviate inflammation, cold, and cough [56], while the fruits of *Hippophae rhamnoides*, the roots of *Bergenia stracheyi*, and *Heracleum candidans* have been reported for gynecological disorders [40]. Similarly, the leaves of *Urtica dioca* were used as a blood purifier and for dermal disorders, the roots of *Rubia cordifolia* for the treatment of boils, ulcers, and dermal issues, and a root paste of *Heracleum candidans* for leucoderma and psoriasis. These uses were different from those recorded in this study (Table 2). Likewise, Ladakhi people use the rhizome of *Bergenia stracheyi* to dissolve kidney stones and for urinary disorders, but it was reported for stomach ulcers in our study. In addition, reported uses of *Aconitum violaceum* (fever), *Carum carvi* (nose pain), *Betula* (burns), and *Chenopodium foliosum* (indigestion) were also different from those currently documented among Baltis and Shina of DNP.

Out of 47 documented species (Table 2), only 14 (29.8%) had been previously reported from surrounding regions. In addition, the vernacular name and medicinal uses of these species were completely different in Baltistan and Ladakh, although both shared many useful species. Vernacular names and utilization may have changed over time, but the species remained constant. This may be linked to the migration of Baltis from India (Ladakh) after the Indo-Pakistan War of 1971. While a number of families settled in Baltistan, their relatives live across the border and they cannot visit each other due to hostile relationship between India and Pakistan. 

### 3.4. Novelty

In the present study, among the 47 reported medicinal plant species, the ethnomedicinal uses of 22 species were documented for the first time from the region. The remaining 26 species (54%) had already been recorded with similar uses. Our findings reveal the current status of the indigenous intercultural variation in plant resource utilization among Balti and Shina communities of the Deosai buffer zone. Among the newly documented species, *Allardia tomentosa*, *Allardia tridactylites*, *Jurinea dolomiaea*, and *Galium boreale* had never been reported in the study for the treatment of human illnesses. Both species of *Allardia* grow at high elevation (above 4000 m), on mountain slopes, in sandy gravel, and along stream edges near glaciers. *Jurinea dolomiaea* and *Galium boreale* are found in moist alpine meadows and subalpine regions of DNP. These species are under anthropogenic pressure due to unsustainable harvesting [44,47]. Unmanaged grazing, trampling, frequent visitations, and off-road driving are particularly destructive activities in the study area, and to date no effective initiatives have been implemented to conserve the plant wealth of this area.

## 4. Conclusions

The current study recorded a considerable amount of traditional ethnomedicinal knowledge and represents the first cross-cultural documentation of medicinal plant uses in the region. A total of 47 medicinal species were documented from both linguistic groups, namely Baltis and Shinas. Leaves were the most commonly used plant parts, followed by flowers, roots, and seeds. A remarkable number of plant species were used in treating gastrointestinal ailments (20 species), followed by skin diseases and respiratory tract disorders (11 species each). Comparative analysis indicated notable differences in plant species used in the traditional health care system by the two groups. Baltis reported a higher number of plant species, which may be due to their earlier settlement in the region. We also recorded quite a large number of differences between the two groups in the medicinal uses of commonly utilized plant species. A comparative analysis with Tibetan ethnobotany indicated that 45% of the medicinal uses were rare or novel, being reported for the first time. In the study area, the local flora is facing serious challenges due to over harvesting and heavy grazing activities. It is critical to underline that the rapid arrival of globalization and modernization could erode traditional knowledge; therefore, we suggest a thorough ethnobotanical investigation to underpin the holistic comparative medical ethnobotany of the entire region before the disappearance of the biocultural heritage of this region, from the deep valleys to the mountaintops. Furthermore, such ethnobotanical surveys could help provide a useful baseline for future conservation programs and will encourage policymakers to endorse the importance of language and culture in maintaining the local environment.

## Figures and Tables

**Table 1:** Characteristics of the targeted localities and study participants.

Language	Village	Elevation (m.a.s.l.)	Number of Interviews	Number of Households	Religion (Faith)	Endogamic/Exogamic Rules	Arrival in the Area	Subsistence Activities
Shina	Sherkuli	3355	12 male/2 female	150	Shia	Endogamic (rarely exogamic)	12th century	Horticulturists and pastoralists
	Sadpara	2350	8 male/2 female	150				
Balti	Dapa	3886	10 male/2 female	180	Shia	Endogamic	Autochthonous	Horticulturists and pastoralists
	Shila	3252	6 male/4 female	70	Nurbakhshi			

**Table 2:** Medicinal plants of Baltis and Shinas in the buffer zone of Deosai National Park, Gilgit-Baltistan, Pakistan.

Name/Family/Voucher Number	Local Name	Habit	PU	DF	Ad	Disease(s) Treated	RSU	RFC
*Aconitum violaceum* Jacquem. ex. Stapf./Ranunculaceae/DNP71	Buma (B/S)	Herb	Root (B/S)	Decoction (B); Powder (S)	Oral (B); Oral and topical (S)	Abdominal pain, flatulence (B); Ringworm, typhoid (S)	No	78.26
*Allardia tomentosa* Decne./Asteraceae/DNP41	Tarkhan (B)	Herb	Flowers (B)	Decoction (B)	Oral (B)	Menstrual cramps, abdominal pain (B)	No	41.30
*Allardia tridactylites* (Kar. and Kir.) Sch. Bip. /Asteraceae/DNP42	Patkanstwa (B)	Herb	Whole plant (B)	Decoction (B)	Oral (B)	Food poisoning (B)	No	28.26
*Allium carolinianum* DC./Amaryllidaceae /DNP35	Refor (B); Kachpauk (S)	Herb	Bulb (B); Leaves (S)	Infusion (B); Boiled leaves (S)	Oral (B/S)	Pharyngitis, bronchitis (B); Constipation (S)	Yes	67.39
*Arnebia benthamii* Wall. ex G. Don/Boraginaceae/DNP51	Thang marsi (B); Kazaban(S)	Herb	Root (B/S)	Mixed with oil (B); Decoction (S)	Topical (B); Oral (S)	Hair tonic (B); Diabetes, pneumonia (S)	No	43.47
*Artemisia brevifolia* Wall./Asteraceae/DNP43	Bursay (B); Zoon (S)	Herb	Aerial parts (B/S)	Decoction (B); Powder (S)	Oral (B/S)	Vermifuges (B); Hypertension (S)	Yes	71.73
*Artemisia scoparia* Waldst. and Kitam./Asteraceae/DNP45	Khobursay (B); Zoon (S)	Herb	Flowers (B); Leaves (S)	Decoction (B); Paste (S)	Oral (B); Oral and topical (S)	Vermifuge, urethritis (B); Ring worm, indigestion (S)	Yes	56.52
*Berberis pseudumbellata* R. Parker/Berberidaceae/DNP48	Skiurbu (B); Ishkeen (S)	Shrub	Root (B); Root, Leaves (S)	Decoction (B/S); Leaves eaten (S)	Oral (B/S)	Hepatitis, diabetes (B/S)	Yes	32.60
*Bergenia stracheyi* (Hook. f. and Thomson) Engl./Saxifragaceae/DNP76	Shapur (B); Sansper (S)	Herb	Root (B/S)	Decoction (B/S)	Oral (B/S)	Stomach ulcer (B); Hepatitis, hypertension (S)	No	60.86
*Betula utilis* D. Don/Betulaceae/DNP49	Jongi (S)	Tree	Bark (S)	Powder (S)	Oral and topical (S)	Back ache, leg pain (S)	Yes	34.78
*Bistorta affinis* (D. Don) Greene/Polygonaceae/DNP67	Chomoi (S)	Herb	Rhizome (S)	Powder	Oral (S)	Diarrhoea, fever (S)	No	54.34
*Carum bulbocastanum* (L.) Koch/Apiaceae/DNP39	Karpho thalay (B); Hayyo (S)	Herb	Seeds (B/S)	Decoction (B/S)	Oral (B/S)	Gastric ulcer (B); Abdominal pain (S)	Yes	43.47
*Carum carvi* L./Apiaceae/DNP38	Naqpo thalay (B); Hayyo (S)	Herb	Seeds (B/S)	Decoction (B); Seeds eaten (S)	Oral (B/S)	Gastric ulcer, gastric trouble (B); Abdominal pain (S)	No	60.80
*Chenopodium foliosum* Asch/Chenopodiaceae/DNP52	Suyaro (S)	Herb	Leaves (S)	Paste (S)	Oral (S)	Oedema, diabetes (C)	No	13.04
*Codonopsis clematidea* (Schrenk) C.B. Clarke/Campanulaceae	Bajo mindoq (B); Tumtaq (S)	Herb	Flowers (B/S)	Infusion (B/S)	Oral (B, S)	Stress relief (B); Male sexual tonic (S)	No	39.13
*Cousinia thomsonii* C.B. Clarke./Asteraceae/DNP40	Charchu (B)	Herb	Flowers (B)	Powder (B)	Topical (B); Oral (S)	Pimples, boils, pustules (B); Constipation (S)	No	43.47
*Delphinium brunonianum* Royle/Ranunculaceae	Makhoting (B); Mahoti (S)	Herb	Flowers (B); Whole plant (S)	Powder + oil (B); Decoction (S)	Topical (B); Oral (S)	Hair tonic (B); Asthma, pneumonia, hair tonic (S)	No	78.26
*Dracocephalum heterophyllum* Benth./Lamiaceae/DNP61	Triba (B)	Herb	Flowers (B)	Decoction (B)	Oral (B)	Abdominal pain, flatulence (B)	Yes	60.86
*Ephedra gerardiana* Wall. ex Stapf/Ephedraceae/DNP55	Chay (B); Soom (S)	Shrub	Fruit, Root (B/S)	Juice, Root paste (B/S)	Oral (B/S)	Tonic and eye pain (B); Asthma, bronchitis (S)	No	52.17
*Fagopyrum esculentum* Moench/Polygonaceae /DNP65	Bro (B)	Herb	Seeds (B)	Powder (B)	Oral (B)	Diabetes, stomach ulcer (B)	Yes	26.08
*Galium boreale* L./Rubiaceae/DNP73	Shatong (B)	Herb	Fruits (B)	Powder (B)	Oral (B)	Hepatitis (B)	No	60.86
*Gentiana olivieri* Griseb/Gentianaceae/DNP57	Tikta (B)	Herb	Flowers (B)	Powder (B)	Oral (B)	Diabetes (B)	Yes	45.34
*Gentiana tianschanica* Rupr. ex Kusn./Gentianceae/DNP59	Palmat (S)	Herb	Flowers, Leaves (S)	Powder (S)	Oral (B)	Blood tonic, hemorrhoids (B)	Yes	43.47
*Heracleum candicans* Wall. ex DC./Apiaceae/DNP37	Ghang (B)	Herb	Root	Powder (B)	Topical (B)	Boils, pimples, pustules (B)	Yes	39.13
*Hippophae rhamnoides* L./Elaeagnaceae/DNP54	Karsokh (B); Buru (S)	Shrub	Fruits (B); Fruits, Seeds (S)	Jam, Powder (B); Paste (S)	Oral and topical (B/S)	Cancer, diabetes, dermatitis (B); Pertussis, cutaneous eruptions (S)	Yes	56.52
*Juniperus excelsa* M. Bieb./Cupressaceae/DNP53	Shukpa (B); Chilli (S)	Tree	Fruits (B/S)	Decoction (B); Dry eaten (S)	Oral (B/S)	Stomach ulcer, fever, diabetes (B); Kidney stones, diabetes (S)	Yes	23.91
*Jurinea dolomiaea* Boiss. /Asteraceae/DNP46	Sathing (B);Gogal Dhoop (S)	Herb	Root (B); Leaves (S)	Paste (B/S)	Topical (B/S)	Fever, cold (B); Wounds, cutaneous eruptions (S)	Yes	52.17
*Mentha royleana* Wall. ex Benth./Lamiaceae /DNP63	Foling (B); Phileel (S)	Herb	Leaves (B/S)	Decoction (B/S)	Oral (B/S)	Hypertension, abdominal pain, obesity (B/S)	Yes	78.26
*Myosotis alpestris* F.W. Schmid/Boraginaceae/DNP50	Mandaqskor (B)	Herb	Flowers (B)	Decoction (B)	Oral (B)	Abdominal pain, fever (B)	Yes	34.78
*Nepeta leucolaena* Benth. ex Hook. f./Lamiaceae/DNP62	Azoomal (B)	Herb	Flowers, Leaves (B)	Decoction (B)	Oral (B)	Gastric ulcer (B)	No	39.13
*Pleurospermum candollei* Benth. ex C.B. Clarke in Hook.f./Apiaceae/DNP36	Shamdun (B)	Herb	Flowers (B)	Decoction (B)	Oral (B)	Stomatitis, constipation, abdominal ache (B)	Yes	41.30
*Ranunculus repens* L./Ranunculaceae/DNP70	Khser mandoq (B)	Herb	Flowers, Leaves (B)	Paste mix with oil (B)	Topical (B)	Pimples, pustules (B)	Yes	21.37
*Rheum australe* D./Polygonaceae/DNP66	Lachu (B); Chontal (S)	Herb	Root (B); Root, Leaves (S)	Powder (B); Decoction (S)	Oral (B/S)	Stomach ulcer (B); Laxative, dyspepsia (S)	Yes	69.56
*Ribes alpestre* Wall. ex Decne./Glossulariaceae/DNP60	Askuta (B); Churkani (S)	Shrub	Fruits (B) Leaves (S)	Eaten fresh, Paste (B/S)	Oral and topical (B/S)	Ringworm, blood tonic (B/S)	No	84.78
*Rosa webbiana* Wall.ex Royle/Rosaceae/DNP72	Siya marfo (B); Shighaye (S)	Shrub	Leaves, Root, Seeds (B/S)	Decoction (B/S)	Oral (B/S)	Jaundice, anemia, tonic (S/B)	No	58.69
*Rubia cordifolia* L./Rubiaceae/DNP74	Zghinoq (B)	Herb	Root (B)	Powder (B)	Oral (B)	Skin inflammation, joint pain (B)	No	32.60
*Rumex hastatus* D. Don./Polygonaceae/DNP68	Churki (S)	Herb	Root (S)	Powder	Topical (S)	Closed bone fractures (S)	No	39.13
*Rumex nepalensis* Spreng./Polygonaceae/DNP69	Hubable (S)	Herb	Root (S)	Powder	Oral and topical (S)	Backache, abdominal pain (S)	Yes	36.95
*Solanum nigrum* L./Solanaceae/DNP77	Drumba Shoghlo (B); Gabeeli (S)	Herb	Seeds (B/S)	Roasted seeds (B/S)	Topical (B/S)	Toothache (B/S)	Yes	52.17
*Swertia petiolata* D. Don./Gentianaceae/DNP58	Brama (B); Mumiri (S)	Herb	Leaves (B); Root (S)	Decoction, Paste, Powder (B/S)	Oral (B); Topical (S)	Hepatitis, pneumonia, dysentery (B); Conjunctivitis (S)	No	71.73
*Tanacetum falconeri* Hook. f./Asteraceae /DNP44	Tyalo (B); Flagyl(S)	Herb	Flower (B/S)	Decoction (B/S)	Oral (B/S)	Body ache, fever (B); Diarrhea, dysentery, abdominal pain (S)	Yes	67.39
*Taraxacum officinale* (L.) Weber ex F.H. Wigg/Asteraceae/DNP47	Khoshmas (B); Guleikasidi (S)	Herb	Whole plant (B); Leaves (S)	Boiled leaves (B/S)	Oral (B/S)	Diabetes, constipation (B/S)	No	43.47
*Thymus linearis* Benth./Lamiaceae/DNP64	Tumburuk (B); Tumuro (S)	Herb	Whole plant (B/S)	Decoction (B/S)	Oral (B/S)	Flatulence, abdominal pain (B); Cough, asthma (S)	Yes	60.86
*Trifolium repens* L./Fabaceae/DNP56	Skabuksuk (B); Chapati (S)	Herb	Flowers (B/S)	Decoction (B/S)	Oral (B/S)	Pneumonia (B); Bronchitis (S)	Yes	56.52
*Urtica dioica* L./Urticaceae/DNP78	Khashoshing (B); Jhoomi (S)	Herb	Aerial parts (B/S)	Boiled leaves (B/S)	Oral (B); Topical (B)	Constipation (B); Pustule, cutaneous eruptions (S)	Yes	43.47
*Verbascum thapsus* L./Scrophulariaceae /DNP75	Apo Tambaku (B); Tamakush (S)	Herb	Flowers, Seeds (B); Leaves, Flowers (S)	Decoction (B/S)	Oral (B/S)	Tonic, labor pain (B); Bronchitis, tuberculosis (S)	No	56.52
*Viola serpens* Wall.ex Ging./Violaceae/DNP79	Skor mindoq (B); Lelo (S)	Herb	Flowers (B/S)	Decoction (B/S)	Oral (B/S)	Abdominal pain, flatulence (B); Bronchitis (S)	No	56.52

B: Baltis, S: Shinas, PU: Part(s) used, DF: Drug formulation, Ad: Administration, RSU: Reports with the same uses.

## Data Availability

All data are included in the manuscript.
